# Effects of exercise modalities on cognitive and muscle function in older adults with cognitive impairment: a systematic review and meta-analysis

**DOI:** 10.1038/s41598-026-48294-9

**Published:** 2026-04-27

**Authors:** Yu Hyeon Choe, Eun-Jeong Cho, Youngju Choi, Dong-Ho Park, Ju-Hee Kang, Hyo-Bum Kwak

**Affiliations:** 1 School of Nursing, Inha University, Incheon, Republic of Korea; 2 Program in Biomedical Science & Engineering, Inha University, Incheon, Republic of Korea; 3 Institute of Sports and Arts Convergence (ISAC), Inha University, Incheon, Republic of Korea; 4 Department of Kinesiology, Inha University, Incheon, Republic of Korea; 5 Department of Pharmacology and Research Center for Controlling Intercellular Communication, Inha University, Incheon, Republic of Korea

**Keywords:** Aged, Cognition, Physical performance, Physical activity, Diseases, Health care, Medical research, Neurology, Neuroscience

## Abstract

This study examined the effects of different exercise interventions on cognitive and muscle function in older adults with mild to moderate cognitive impairment or dementia. Randomized controlled trials published up to May 2025 were identified from four databases (Cochrane Library, PubMed, EMBASE, and Web of Science). Aerobic, resistance, and combined exercise interventions were included. Study quality was assessed using the Cochrane RoB 2 tool, and random-effects meta-analyses were conducted. Fourteen studies involving 1,097 participants were included. The meta-analysis indicated that aerobic exercise was mainly associated with improvements in cognitive outcomes, whereas resistance exercise primarily enhanced muscle strength and physical performance. Interventions combining aerobic and resistance components showed benefits across both cognitive and muscle function domains. Although the included studies were few and heterogeneous, the overall pattern suggests potential modality-specific effects, whereby aerobic exercise may support cognitive function, resistance exercise may enhance muscle strength and physical performance, and combined exercise may provide benefits for both cognition and muscle function in older adults with mild to moderate cognitive impairment or dementia. However, the certainty of evidence ranged from low to very low, and these findings should therefore be interpreted with caution. These findings highlight the value of selecting exercise modalities based on targeted functional goals.

*PROSPERO registration*: CRD420251051222

**Supplementary Information:**

The online version contains supplementary material available at 10.1038/s41598-026-48294-9.

## Introduction

With the growth in the aging population worldwide, the prevalence of mild to moderate cognitive impairment and dementia has increased significantly, posing major challenges for public health and geriatric care^1^. Older adults with cognitive impairment engage in less physical activity, even at low intensities, compared with cognitively healthy individuals^2^. This reduced activity level, largely driven by cognitive deficits, has been reported to accelerate declines in muscle function, such as muscle strength, gait, balance, and motor coordination^3,4^. These declines are hallmarks of ‘sarcopenia’, a condition characterized by the progressive loss of muscle mass, strength, and physical performance^5^. Sarcopenia frequently co-occurs with cognitive impairment as aging progresses. Indeed, meta-analytic evidence indicates that individuals with sarcopenia have markedly increased odds ratios, approximately two to three times higher, for experiencing cognitive impairment compared to those without sarcopenia^6,7^. The coexistence of cognitive and muscle dysfunction establishes a vicious feedback loop, exacerbating age-related decline, increasing vulnerability to frailty and functional dependence^8,9^. Given the bidirectional relationship between cognitive decline and sarcopenia, interventions targeting only one domain may be insufficient to prevent functional deterioration^8,9^. Therefore, strategies that account for the interconnected nature of cognitive and muscle dysfunction are particularly warranted in this population.

Currently, the cognitive benefits of pharmacological treatments remain limited, whereas exercise has emerged as a non-pharmacological strategy that not only supports brain health but also improves muscle strength, mobility, and physical function in older adults. Growing evidence suggests that various exercise modalities, including aerobic, resistance and multicomponent exercise training, can significantly enhance cognitive outcomes such as memory and executive function, as well as physical performance in older adults^10,11^. In fact, both the World Health Organization (WHO) and the American College of Sports Medicine (ACSM) emphasize the importance of physical activity by recommending that older adults engage in at least 150 min of moderate-intensity aerobic activity per week and perform muscle-strengthening exercises on two or more days^12,13^. Notably, a recent meta-analysis demonstrated significant improvements in both cognitive and gait performance following exercise interventions in individuals with mild cognitive impairment. However, many older adults with cognitive impairment are unable to meet these recommended levels of physical activity due to cognitive, motivational, and physical limitations^2,14^. These factors contribute to low adherence to physical activity guidelines^15^, emphasizing the need for research that identifies the most suitable exercise guidelines specifically tailored for individuals with cognitive impairment. However, it remains unclear whether specific exercise modalities differentially influence cognitive and muscle-related outcomes in this population, limiting the ability to formulate targeted exercise prescriptions.

Recently, several network meta-analyses have examined the effects of exercise interventions in older adults with cognitive impairment. Although some studies suggested that multicomponent exercise may yield greater cognitive benefits, direct and balanced comparisons between aerobic, resistance, and combined exercise modalities remain limited^16^. Furthermore, most prior syntheses have primarily focused on cognitive outcomes without concurrently evaluating muscle strength or physical performance^17,18^. As a result, it remains unclear whether different exercise modalities exert domain-specific or complementary effects across cognitive and muscle-related functions. This gap limits the development of targeted, modality-specific exercise recommendations for older adults with cognitive impairment.

To address these gaps in the literature, the present systematic review and meta-analysis compared the effects of aerobic, resistance, and combined exercise interventions on both cognitive and muscle function in older adults with mild to moderate cognitive impairment or dementia. By examining modality-specific and complementary effects across domains, this study sought to provide evidence to support more precise and tailored exercise recommendations for this population. The primary outcome was cognitive function, and secondary outcomes included muscle strength and physical performance.

## Methods

### Protocol registration

The protocol of this systematic review was registered with the International Prospective Register of Systematic Reviews (PROSPERO)^19^, under the registered identification number CRD420251051222 (available from: https://www.crd.york.ac.uk/PROSPERO/view/CRD 420251051222).

### Search strategy

A comprehensive search was conducted in the following databases: Cochrane Library, PubMed, EMBASE, and Web of Science, from their inception to May 2025 to identify eligible studies. Search terms were adapted for each database. Comprehensive literature searches were conducted using Medical Subject Headings (MeSH) terms and free text words, including “cognitive dysfunction”, “dementia”, “exercise”, “cognition”, and “physical performance”. The detailed Cochrane Library search strategy is provided in Supplementary Information File (Table S1).

### Eligibility criteria

This study is based on the Preferred Reporting Items for Systematic Reviews and Meta-analyses (PRISMA) guidelines^20^. The articles included in this systematic review met the following inclusion criteria: (1) Participants: older adults aged 60 years or older with mild to moderate cognitive impairment or dementia who had received a clinical diagnosis of cognitive impairment or dementia. To enhance clinical coherence and population comparability, this review focused on cognitive impairment associated with aging, excluding studies primarily involving cognitive impairment attributable to vascular pathology or traumatic brain injury. While mild to moderate cognitive impairment and dementia represent distinct clinical stages, they are increasingly recognized as part of a continuous spectrum of neurodegenerative decline, sharing overlapping pathophysiological mechanisms and functional deficits^21^. Consequently, participants in the included trials often reflected this clinical continuum rather than strictly segregated diagnostic categories; (2) Intervention: aerobic exercise (e.g., walking, cycling), resistance exercise (e.g., strength training, weightlifting, elastic band training), and combined exercise interventions (e.g., multicomponent or concurrent programs integrating aerobic and resistance modalities); (3) Comparison: control group with no exercise intervention (e.g., health education, counseling); (4) Outcome: cognitive and muscle function. Muscle function was further categorized into two domains: muscle strength and physical performance. Muscle strength was defined as the ability of a muscle to generate force (e.g., handgrip strength, knee extensor strength), whereas physical performance reflects the capacity to perform functional tasks involving mobility, balance, and coordination (e.g., gait speed, balance tests, composite measures such as the Short Physical Performance Battery); (5) Study design: Randomized Controlled Trials (RCTs) only, to ensure high-quality evidence; (6) Published in English; (7) Full-text articles published in peer-reviewed journals.

### Study selection

According to the inclusion criteria, all studies were imported into the reference manager software (EndNote X20 for Windows; Clarivate Analytics, Philadelphia, Pennsylvania, USA) to exclude duplicate studies. Two reviewers (Y.H.C. and E.J.C.) independently screened all identified studies for relevance. A pilot screening was conducted on 70 randomly selected studies (35 in each of two rounds), and inter-rater agreement was assessed using Cohen’s kappa (κ = 0.53), indicating moderate agreement. Subsequently, the same reviewers evaluated all titles and abstracts and conducted full-text screening. During the full screening phase, regular discussions were conducted to ensure consistency, and any disagreements were resolved by consensus.

### Data extraction

The two researchers (Y.H.C., E.J.C.) independently extracted data using a data extraction sheet in Microsoft Excel (Microsoft Corporation, Redmond, Washington, USA), organizing the following elements: author, publication year, country, type of cognitive impairment, gender, sample size, mean age, the characteristics of the exercise interventions (type, frequency, minutes per session, intensity, duration), and measured outcomes. To enhance data extraction accuracy, a pilot test was conducted on three articles, and the template was refined by consensus.

### Quality assessment

We assessed the quality of included studies using the revised Cochrane risk-of-bias tool for randomized trials (RoB2)^22^. The RoB2 provides a checklist tailored for RCTs designs and comprises five domains: (1) randomization process, (2) deviations from intended interventions, (3) missing outcome data, (4) measurement of the outcome, and (5) selection of the reported result. Each domain is rated using one of the following responses: “Yes”, “Probably yes”, “Probably no”, “No”, or “No information”. Overall appraisal was categorized as “Low”, “Some concerns”, or “High”. Two independent researchers (Y.H.C. and E.J.C.) conducted the quality assessment, and any discrepancies were resolved by a third reviewer (H.B.K.).

### Certainty of evidence

The certainty of evidence for each outcome was assessed using the GRADE (Grading of Recommendations Assessment, Development and Evaluation) approach^23^. While evidence from randomized controlled trials was initially rated as high certainty, it was downgraded based on five domains: risk of bias, inconsistency, indirectness, imprecision, and publication bias^24^. The overall certainty of evidence was rated as high, moderate, low, or very low, with the results summarized in the Summary of Findings table (Supplementary Information Table S2).

### Data analysis

In this study, a qualitative synthesis was conducted as part of a systematic literature review to thoroughly examine the characteristics of the included studies. Extracted data were structured and analyzed in accordance with the objectives of each analytical procedure. The findings from the qualitative synthesis were combined with quantitative results to deliver a holistic evaluation of the effects of exercise interventions on cognitive and muscle function. In parallel, quantitative synthesis was performed using the Review Manager 5.4.1 software (RevMan, Cavendish Square, London, United Kingdom). Effect sizes were calculated as standardized mean differences (SMD) with 95% confidence intervals (CIs). Given the anticipated clinical and methodological heterogeneity across studies—including differences in diagnostic categories, intervention characteristics, and outcome measures—a random-effects model was applied to account for between-study variability and to provide more conservative pooled estimates. In addition, potential sources of heterogeneity were explored qualitatively through examination of participant characteristics, intervention dose (frequency, duration, and intensity), and outcome measurement tools. Results of the meta-analyses were presented using forest plots. For multi-arm trials, intervention arms were included only in separate modality-specific meta-analyses. Because shared control groups were not included more than once within any single pooled comparison, double-counting was avoided. Heterogeneity was assessed using the I^2^ statistic. We took I^2^ values of 0%, 25%, 50%, and 75% as representing no, low, moderate, and high heterogeneity, respectively^25^. We did not present a funnel plot in this study because fewer than 10 studies were available for each variable, the minimum generally required for such analysis^26^. *P* < 0.05 was considered statistically significant.

## Results

### Study identification

**Fig. 1:**
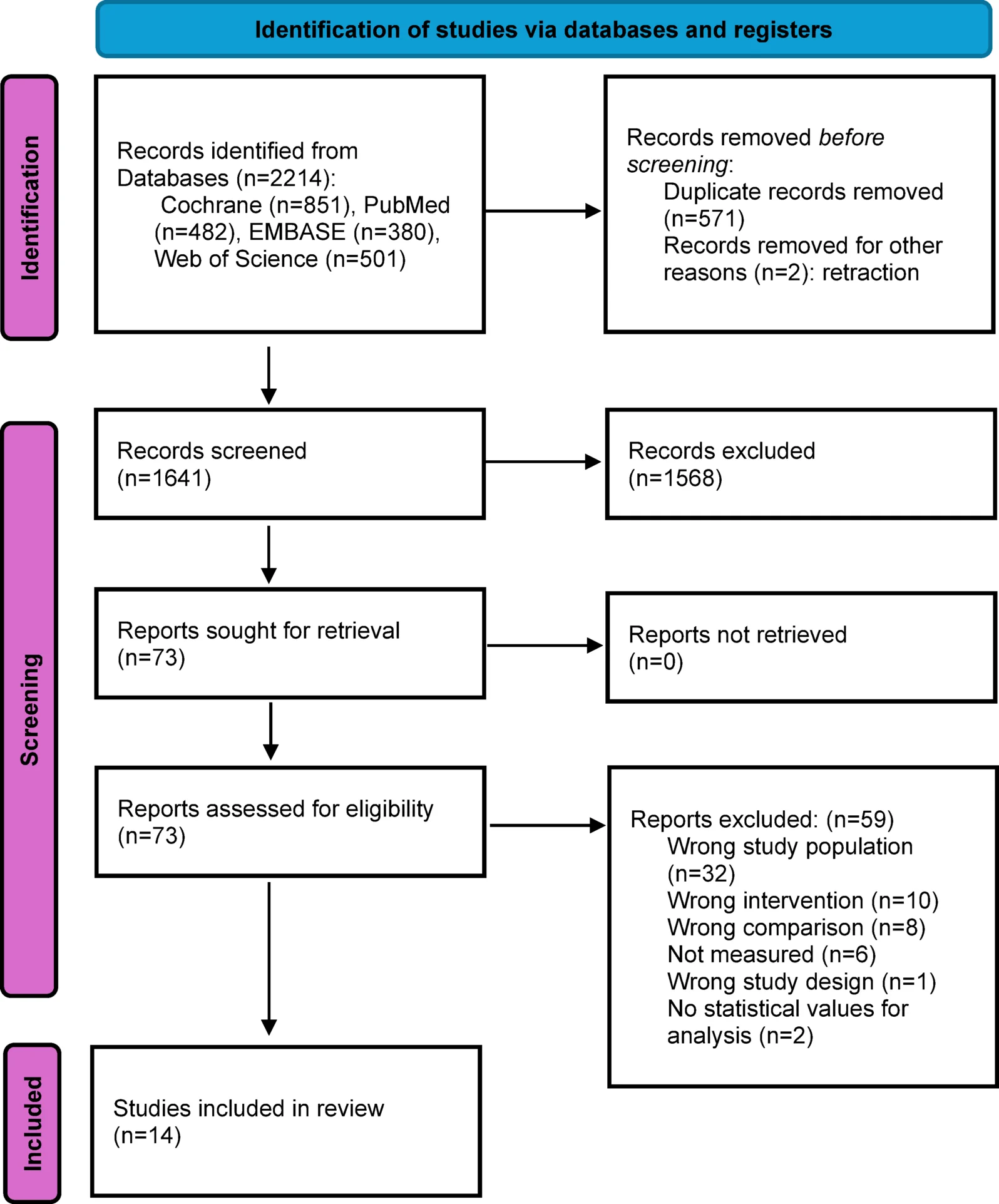
Selection process of included studies according to the PRISMA (Preferred Reporting Items for Systematic Reviews and Meta-Analysis) guideline.

We retrieved 2,214 articles from 4 databases. After 571 duplicates and 2 retracted articles were removed, 1,641 records remained for screening. Following title and abstract screening, 1,568 articles were excluded, and two researchers independently assessed 73 full-text articles for eligibility. Of these, 57 articles did not meet the inclusion criteria. Two additional studies met the inclusion criteria but did not report sufficient statistical data for effect size calculation; attempts were made to contact the authors, but no response was received, and thus these studies were excluded. Ultimately, 14 studies were included in the review following the systematic screening process (Fig. 1).

### Study characteristics

This systematic review included 14 RCTs, with detailed characteristics presented in Table 1. Altogether, 1,097 participants were enrolled across these studies. Among the included studies, five implemented aerobic exercise intervention (AEI)^27–31^, four involved resistance exercise intervention (REI) alone^31–34^, and six applied a combined exercise intervention (CEI)^35–40^. In the study by Huang^31^, both AEI and REI were conducted separately and were therefore counted in both categories. Across all 14 included studies, the duration of exercise interventions ranged from 8 weeks to 15 months, five studies^28,33,35–37^ implemented 12-week intervention period. The frequency of exercise interventions ranged from two days per week to daily sessions. Session durations were categorized according to the type of exercise intervention as follows: AEIs ranged from 15 to 60 min per session, REIs ranged from 30 to 90 min, and CEIs were conducted for 30 to 60 min per session. In terms of intensity, AEIs were performed at moderate intensity in four studies^28–31^ and at mild intensity in one study^27^. All four REI studies reported moderate intensity. Among the six CEI studies, five met at least moderate intensity for both aerobic and resistance components^37–40^. In one study^35^, AEI was conducted at mild intensity, while the intensity of the REI was not clearly specified. For cognitive function, the most frequently used measurement was the Mini-Mental State Examination (MMSE)^27,30,32,34,37,39,40^, followed by the Montreal Cognitive Assessment (MoCA)^31,33,35,36,40^. Muscle function was evaluated in terms of muscle strength and physical performance. Muscle strength was measured using the Sit-to-Stand Test^29,33–35,40^, handgrip strength^36^, and knee extensor muscle strength^31^. Physical performance was assessed using tools such as the Short Physical Performance Battery (SPPB)^31,34,36^, Timed Up and Go (TUG) test^27,28,35,37^, the 6-Minute Walk Test (6MWT)^30,31^, and walking speed^28,33,38^.

**Table 1:** Characteristics of included studies.

Author (year)	Country	Cognitive impairment type	Gender	Sample size		Age (year)		Intervention type	Days per week	Minutes per session	Intensity	Duration	Outcomes
				EG	CG	EG	CG						
Akbuga Koc^35^	Turkey	AD	Male, female	20	20	78.9 ± 6.9	76.7 ± 6.9	Combined	2	60	AEI=Mild REI = 12 repetitions^*^	12 weeks	Muscle strength, physical performance, cognition
Cancela^27^	Spain	Dementia	Male, female	73	116	82.90 ± 7.42	80.63 ± 8.32	Aerobic	7	Minimum 15	Mild	15 months	Physical performance, cognition
Casas-Herrero^36^	Spain	MCI or mild dementia	Male, female	88	100	84.2 ± 4.8	84.0 ± 4.8	Combined	5	60	AEI=Moderate REI=Moderate	12 weeks	Muscle strength, physical performance, cognition
Donnezan^28^	France	MCI	NR	21	15	77.1 ± 1.44	79.2 ± 4	Aerobic	2	60	Moderate	12 weeks	Physical performance, cognition
de Oliveira Silva^37^	Brazil	MCI	Male, female	14	14	71.85 ± 5.69	78.20 ± 5.26	Combined	2	60	AEI=Moderate REI=Moderate	12 weeks	Physical performance, cognition
		AD	Male, female	14	14	81.22 ± 8.88	77.54 ± 8.05						
Kemoun^38^	France	AD	Male, female	20	18	82.0 ± 5.8	81.7 ± 5.1	Combined	3	60	AEI=Moderate to vigorous REI=Moderate to vigorous	15 weeks	Physical performance, cognition
Kwak^32^	South Korea	Dementia	Female	15	15	79.67 ± 6.64	82.27 ± 7.09	Resistance	2 ~ 3	30 ~ 60	Mild to moderate	12 months	Muscle strength, physical performance, cognition
Langoni^39^	Brazil	MCI	Male, female	30	30	72.6 ± 7.8	71.9 ± 7.9	Combined	2	60	AEI=Moderate to vigorous REI=Moderate	24 weeks	Muscle strength, physical performance, cognition
Law^29^	China	MCI	Male, female	37	36	77.35 ± 6.66	74.14 ± 7.53	Aerobic	NR	60	Moderate	8 weeks	Muscle strength, physical performance, cognition
Levinger^33^	Australia	Mild to moderate dementia	Male, female	9	8	83.3 ± 7.5	87.5 ± 3.0	Resistance	2	60 ~ 90	Moderate	12 weeks	Muscle strength, physical performance, cognition
Li^40^	China	MCI	Male, female	45	45	Above 60	Above 60	Combined	5	30	AEI=Moderate REI=Moderate	6 months	Physical performance, cognition
Mak^34^	Australia	Mild to moderate cognitive impairment or dementia	Male, female	76	72	86.0	87.2	Resistance	2	60	Moderate	24 weeks	Muscle strength, physical performance, cognition
Venturelli^30^	Italy	AD	Male, female	12	12	83 ± 6	85 ± 5	Aerobic	4	30	Moderate	24 weeks	Physical performance, cognition
Huang^31^	China	MCI	Male, female	36	36	71.11 ± 7.65	73.86 ± 7.50	Aerobic	3 ~ 5	30 ~ 50	Moderate	6 months	Muscle strength, physical performance, cognition
			Male, female	36		71.75 ± 6.71		Resistance	2	50	Moderate	6 months	Muscle strength, physical performance, cognition

AD=Alzheimer’s disease; AEI=aerobic exercise intervention; EG=experimental group; CG=control group; MCI=mild cognitive impairment; NR = not reported; REI=resistance exercise intervention. ^*^ The intensity was not clearly specified.

### Quality appraisal

**Fig. 2:**
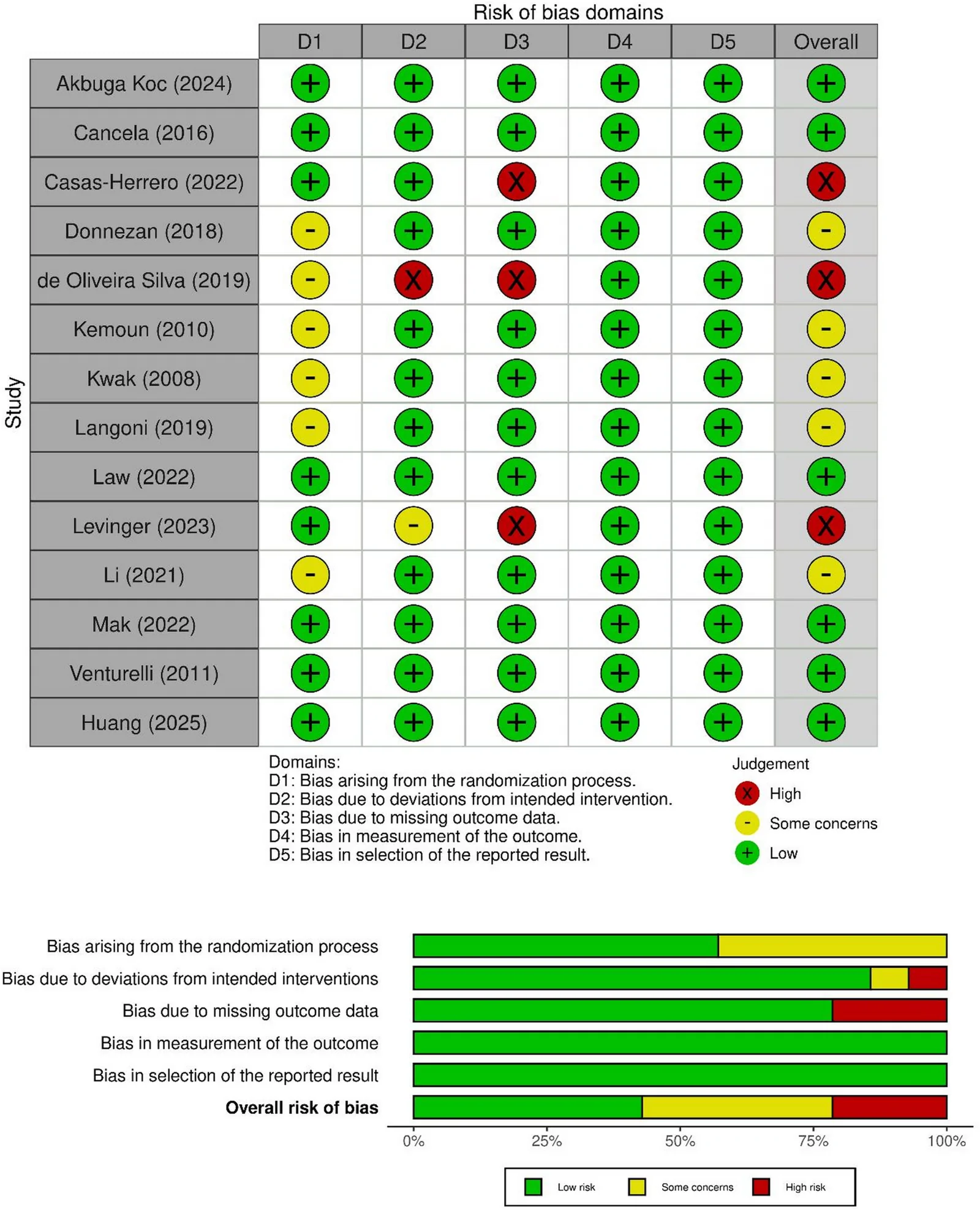
Quality of included studies.

The risk of bias was assessed using the RoB 2 tool, and the results of the quality assessment were visualized using the Risk-of-Bias VISualization (robvis) tool to provide a detailed summary of potential biases in the included studies^41^. The quality appraisal results of the included studies were presented in Fig. 2. Among the 14 included studies, the overall judgment was rated as low in 6 studies, as having some concerns in 5, and as high in the remaining 3. All studies were assessed as low risk in the domains of outcome measurement and selective reporting. Six studies reported some concerns regarding the randomization process. Three studies were judged to have a high risk of bias, primarily due to missing outcome data^33,36,37^. Additionally, risk of bias based on intention-to-treat (ITT) and per-protocol (PP) analyses was provided in the Supplementary Information (Figure S1).

### Meta-analysis of cognitive function by exercise intervention types

**Fig. 3:**
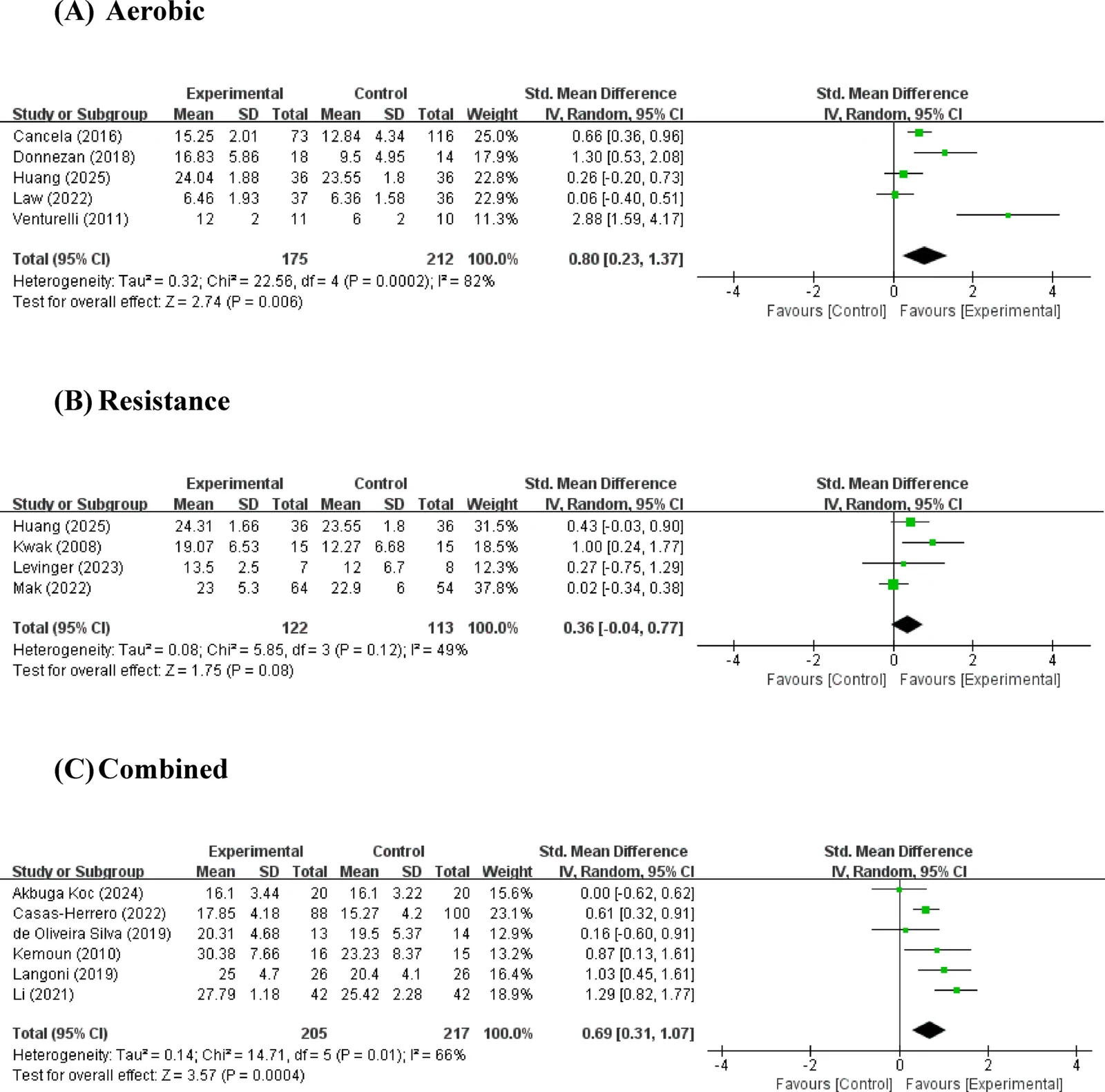
Effect of exercise interventions on cognitive function.

Figure 3 presents the results of a meta-analysis investigating the effect of exercise intervention types on cognitive function in older adults with mild to moderate cognitive impairment or dementia. AEI showed a statistically significant improvement in cognitive function compared to control groups (6 studies, SMD = 0.80, 95% CI = 0.23 to 1.37, *P* = 0.006). Heterogeneity was high variability (I² = 82%), indicating differences across studies. In contrast, REI did not demonstrate a significant effect on cognitive outcomes (4 studies, SMD = 0.36, 95% CI = − 0.04 to 0.77, *P* = 0.08). A CEI was associated with a significant effect on cognitive function (6 studies, SMD = 0.69, 95% CI = 0.31 to 1.07, *P* = 0.0004), with moderate heterogeneity observed across studies (I² = 66%).

### Meta-analysis of muscle function by exercise intervention types

**Fig. 4:**
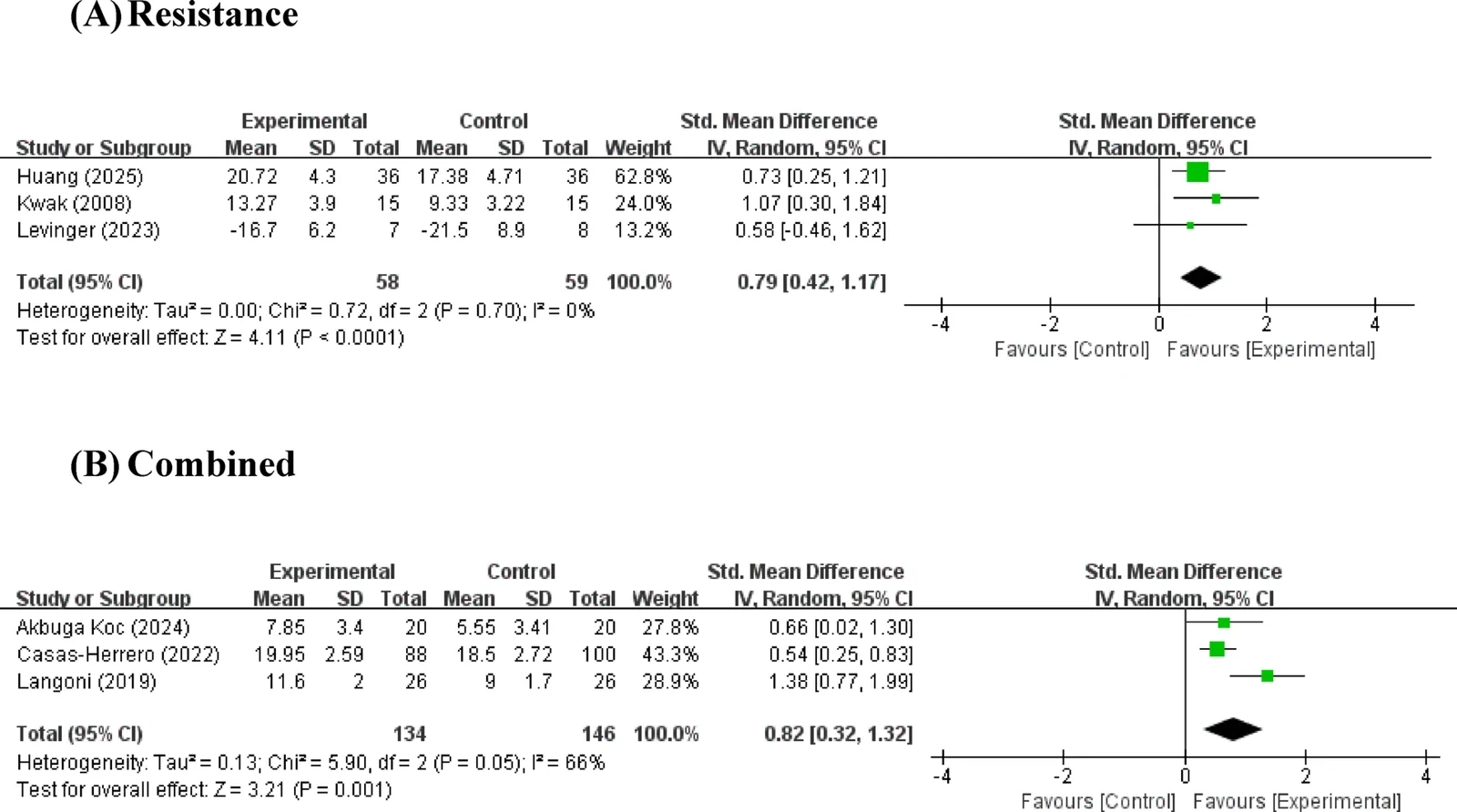
Effect of exercise interventions on muscle strength.

**Fig. 5:**
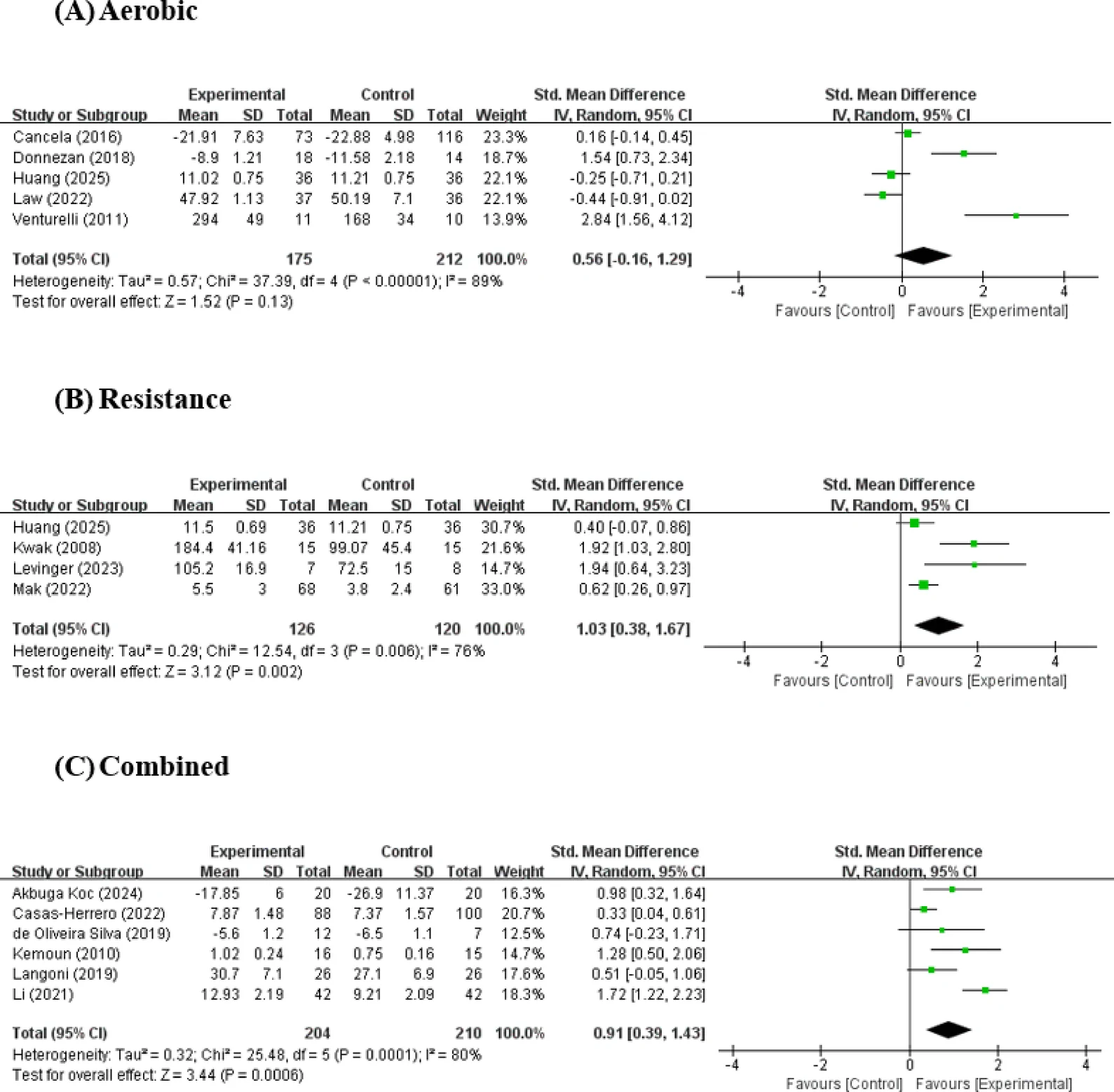
Effect of exercise interventions on physical performance.

This meta-analysis was conducted to evaluate the effectiveness of exercise interventions on muscle function, analyzed as muscle strength and physical performance. The findings are presented in Figs. 4 and 5.

#### Muscle strength

The effect of exercise interventions on muscle strength was quantitatively assessed. Figure 4 presents the results for REI and CEI. Only two studies^29,31^ assessed muscle strength; therefore, a meta-analysis could not be conducted for this exercise type. In the study by Law et al.^29^, muscle strength in the intervention group significantly improved compared with the control group. In contrast, the study by Huang et al.^31^ reported a decrease in muscle strength in some measures and no significant change in others, indicating inconsistent findings across studies. REI significantly improved muscle strength compared with the control group (3 studies, SMD = 0.79, 95% CI = 0.42 to 1.17, *P* < 0.0001). No heterogeneity was observed among the included studies (I² = 0%). CEIs also showed a large effect on improving muscle strength (3 studies, SMD = 0.82, 95% CI = 0.32 to 1.32; *P* = 0.001). The level of heterogeneity was moderate (I² = 66%).

#### Physical performance

A pooled analysis evaluated the effects of different exercise intervention modalities on physical performance, as shown in Fig. 5. AEI did not result in a statistically significant gain in physical performance (5 studies, SMD = 0.56, 95% CI = − 0.16 to 1.29, *P* = 0.13). For REI, a significant positive effect on physical performance was observed in the experimental group compared with the control group (4 studies, SMD = 1.03, 95% CI = 0.38 to 1.67, *P* = 0.002). Heterogeneity across these studies was considerable (I² = 76%). The effect of CEI was examined, and participants in the experimental group demonstrated significant improvements compared to those in the control group (6 studies, SMD = 0.91, 95% CI = 0.39 to 1.43, *P* = 0.0006), with high heterogeneity observed (I² = 80%). The high level of heterogeneity was attributed to substantial variability among the included studies.

### Certainty of evidence

The GRADE assessment indicated that the certainty of evidence for the examined outcomes ranged from low to very low. Detailed downgrading criteria and the Summary of Findings are presented in Supplementary Information Table S2.

## Discussion

This systematic review and meta-analysis was conducted to evaluate the effects of different types of exercise interventions on cognitive and muscle function in older adults with mild to moderate cognitive impairment or dementia. Overall, the findings suggest potential modality-specific patterns of benefit: AEIs were mainly associated with improvements in cognitive outcomes, REIs with gains in muscle strength and physical performance, and CEIs with improvements across both cognitive and muscle function.

An examination of the characteristics of the CEIs included in this meta-analysis showed that the frequency of sessions ranged from 2 to 5 times per week. The duration of each session was 60 min in all but one study, most exercise interventions were performed at a moderate intensity, and the intervention period ranged from 12 weeks to 6 months. Among the included studies, three implemented CEIs twice a week^35,37,39^. This supports prior findings suggesting that a lower weekly frequency may be more suitable for older adults with mild to moderate cognitive impairment or dementia, considering the potential for rapid changes in health status over a relatively short period^42^. In contrast, the study by Li et al.^40^ implemented 30-minute sessions conducted five times per week for six months and reported significant improvements in both cognitive and muscle function. This result was interpreted as reflecting an intervention strategy that reduced intensity and session duration, and strategically distributed time across different exercise modalities, tailored to the functional limitations of older adults with cognitive decline^43^. Taken together, CEIs were generally associated with benefits across domains^44^, but variability in baseline functional status, participant characteristics, intervention components, and outcome measures likely contributed to between-study heterogeneity; therefore, current evidence should be considered suggestive rather than conclusive. Despite the existence of general physical activity guidelines provided by organizations such as the WHO and ACSM^12,13^, there remains a lack of standardized exercise protocols specifically tailored for older adults with cognitive impairment. Therefore, further efforts to develop and validate evidence-based, standardized exercise guidelines tailored to this population are warranted.

An overview of the AEIs included in this analysis follows. AEIs were performed 2 to 7 times per week, with most sessions lasting at least 30 min. The intensity ranged from mild to moderate, and the duration of interventions varied from 8 weeks to 6 months. Several studies reported that the total volume of aerobic activity did not meet the recommendations set by the ACSM. This may be attributed to the limited endurance capacity of individuals with cognitive impairment^45^. Aerobic activities such as walking and cycling also demand balance, coordination, and sustained attention, placing additional cognitive and physical strain^46^. Nevertheless, participants in the experimental groups demonstrated significant improvements in cognitive function compared with control groups. These outcomes may be attributed to mechanisms such as increased cerebral blood flow and enhanced neuroplasticity, aligning with previous findings that support the role of aerobic exercise in cognitive enhancement among older adults^47–49^.

All REI studies complied with ACSM guidelines^13^ recommending at least two sessions per week and progressive overload. Participants demonstrated improvements in muscle strength and physical performance. From a cognitive perspective, three of the four studies included in the meta-analysis^31,33,34^ did not report statistically significant improvements in cognitive function following resistance exercise interventions, despite implementing training programs at a frequency of two sessions per week, with session durations ranging from 50 to 90 min over intervention periods of at least 12 weeks. In contrast, the study by Busse et al.^50^ observed significant cognitive improvements following a resistance training program conducted twice weekly for 60 min over a prolonged period of nine months. Taken together, these findings indicate that the current evidence base remains limited with respect to cognitive outcomes following resistance exercise. Although longer-duration programs may show potential, the small number of available studies and modest sample sizes necessitate cautious interpretation.

Among the various outcomes, the AEI–cognition analysis demonstrated the most robust evidence with moderate certainty, despite substantial heterogeneity (I² = 82%). This variability likely reflects diverse diagnostic categories—mild cognitive impairment, dementia, and Alzheimer’s disease—and inconsistent intervention dose (frequency, intensity, and duration). Although formal subgroup analyses were limited by the small number of studies, visual inspection of forest plots revealed no systematic clustering by diagnostic category or assessment tool (e.g., MMSE vs. MoCA). This suggests that the heterogeneity stems from broader clinical diversity and baseline severity gradations rather than instrument-specific biases, supporting potential benefits of AEI across the cognitive decline spectrum. In contrast, the evaluation of AEI effects on muscle strength was constrained by insufficient evidence, with only two studies providing inconsistent data. As suggested by the lack of statistical significance here, these results should be interpreted as a reflection of a limited evidence base rather than a definitive lack of efficacy. Similarly, physical performance analyses across AEI, REI, and CEI showed marked heterogeneity (I² = 76–89%), primarily driven by the use of functionally distinct assessment tools (e.g., gait speed vs. composite scales) and varying baseline mobility. Participants with lower baseline performance likely exhibited greater responsiveness compared to those with preserved capacity, contributing to high between-study variance. Overall, while our pooled estimates suggest potential clinical benefits, the high heterogeneity across all modalities reinforces that exercise responses are highly individualized, necessitating cautious interpretation within a standardized prescription framework.

Importantly, to further examine the potential influence of studies rated as high risk of bias, sensitivity analyses were conducted excluding Casas-Herrero et al.^36^ and de Oliveira Silva et al.^37^. The pooled effects for combined exercise interventions remained statistically significant for both cognitive outcomes (SMD = 0.81, 95% CI = 0.25 to 1.38) and physical performance (SMD = 1.12, 95% CI = 0.55 to 1.70), indicating that the overall findings were not driven by higher-risk studies. In the resistance exercise analyses, exclusion of the study rated as high risk of bias resulted in a modest attenuation of the pooled estimate; however, the direction and statistical significance of the effect were preserved, suggesting that the observed benefits were not solely attributable to higher-risk evidence. Notably, the increase in pooled effect size following exclusion of higher-risk studies may reflect reduced variance rather than a true amplification of effect and should not be interpreted as evidence of stronger efficacy.

From a clinical perspective, these findings support a tailored approach, suggesting that exercise modalities can be strategically selected based on functional goals: AEIs for cognitive outcomes, REIs for muscle strength, and CEIs for broader, integrative benefits^51^. However, translating these findings into routine care requires careful consideration of feasibility and resources. While AEI may be adaptable to community walking programs, REI can be effectively delivered using simple equipment (e.g., elastic bands) in home or primary care settings. Conversely, CEI may require more structured coordination, trained personnel, and greater resource allocation to ensure safe implementation. Across all modalities, supervision, adherence, caregiver involvement, and fall risk remain pivotal, consistent with consensus guidelines emphasizing individualized prescriptions for older adults with cognitive decline^12,43^. Given the reported adherence challenges in this population^42^, aligning these interventions with public health policies for healthy aging necessitates pragmatic implementation designs. Future research should focus on how these modality-specific benefits can be sustainably integrated into existing healthcare infrastructures to maximize real-world impact.

To further contextualize these findings, the certainty of evidence was assessed using the GRADE approach. The GRADE assessment indicated that the certainty of evidence ranged from low to very low across outcomes, primarily due to substantial heterogeneity, methodological limitations, and imprecision associated with small sample sizes. These findings suggest that, although exercise interventions appear to show beneficial trends across cognitive and muscle function domains, the current evidence base remains limited^43^. Therefore, the results should be interpreted cautiously, and future well-designed trials with standardized outcome measures and larger samples are needed to strengthen the certainty of the evidence.

This study has limitations. The number of studies included in the meta-analysis was limited; therefore, the pooled estimates should be interpreted cautiously, which may restrict the generalizability of the findings. Substantial heterogeneity was observed in some analyses, likely reflecting differences in intervention characteristics, outcome measures, and participant characteristics. In particular, the absence of stratified analyses by the severity of cognitive impairment (e.g., distinguishing between mild cognitive impairment and dementia) limits our ability to determine stage-specific treatment responses, largely due to the limited number of trials available for each diagnostic category. Additionally, the use of various cognitive assessment tools with differing psychometric sensitivities (e.g., MMSE vs. MoCA) may have contributed to variability in effect estimates across studies. The restriction to English-language publications may also have introduced language bias and limited the comprehensiveness of the evidence base. Overall, these limitations highlight the need for future high-quality trials with more standardized intervention protocols, outcome measures, and larger sample sizes to strengthen the evidence base across the spectrum of cognitive decline.

## Conclusion

In conclusion, this systematic review and meta-analysis suggests that exercise interventions may exert domain-specific and complementary effects on cognitive and muscle function in older adults with mild to moderate cognitive impairment or dementia. Aerobic exercise interventions were associated with improvements in cognitive function, whereas resistance exercise interventions demonstrated beneficial effects on muscle strength and physical performance. Combined exercise interventions integrating aerobic and resistance components showed improvements across cognitive function, muscle strength, and physical performance, indicating their potential as a comprehensive strategy for addressing the multidimensional functional decline associated with cognitive impairment. However, the certainty of evidence ranged from low to very low across most outcomes, and therefore these findings should be interpreted with caution. These findings underscore the potential clinical relevance of integrative exercise approaches and emphasize the need for future high-quality trials to establish standardized, evidence-based exercise guidelines tailored to this vulnerable population.

## Supplementary Information

Below is the link to the electronic supplementary material.

Supplementary Material 1

## Data Availability

All data generated or analyzed during this study are included in this published article (and its supplementary information file).
