# Supplementary material for: Effects of exercise modalities on cognitive and muscle function in older adults with cognitive impairment: a systematic review and meta-analysis
Source: Sci Rep. 2026 Apr 27;16:19319. doi: 10.1038/s41598-026-48294-9 (PMC13284201; doi:10.1038/s41598-026-48294-9)
Supplement: Supplementary file 1 — Supplementary Material 1 [file 41598_2026_48294_MOESM1_ESM.docx]

**Supplementary information**

Effects of exercise modalities on cognitive and muscle function in older adults with cognitive impairment: a systematic review and meta-analysis

**Table S1**. Search strategy used in Cochrane Library

**Table S2**. Summary of findings and GRADE certainty of evidence assessment

**Figure S1**. Risk of bias assessment according to ITT and PP analyses

**Table S1.** Search strategy used in Cochrane Library

| #1 MeSH descriptor: [Alzheimer Disease] explode all trees 5619  #2 alzheimer* 17024  #3 MeSH descriptor: [Dementia] explode all trees 9961  #4 dementia* 32371  #5 MeSH descriptor: [Cognitive Dysfunction] explode all trees 4584  #6 {OR #1-#5} 38935  #7 MeSH descriptor: [Exercise] explode all trees 40107  #8 aerobic training 13427  #9 aerobic exercise 19997  #10 MeSH descriptor: [Resistance Training] explode all trees 5994  #11 resistance exercise 21164  #12 strength training 25606  #13 MeSH descriptor: [Weight Lifting] explode all trees 1141  #14 weight training 15281  #15 physical activit* 78302  #16 {OR #7-#15} 137256  #17 muscle mass 16728  #18 muscle strength 33607  #19 MeSH descriptor: [Physical Functional Performance] explode all trees 660  #20 physical performance 31180  #21 sarcopenia assessment 811  #22 {OR #17-#21} 66784  #23 MeSH descriptor: [Cognition] explode all trees 16505  #24 cognitive function 32602  #25 {OR #23-#24} 44227  #26 MeSH descriptor: [Randomized Controlled Trial] explode all trees 34  #27 randomi* controlled trial 1163298  #28 RCT 54360  #29 {OR #26-#28} 1170707  #30 #6 AND #16 AND #22 AND #25 AND #29 851 |
| --- |

**Table S2**. Summary of findings and GRADE certainty of evidence assessment

| **Certainty assessment** | | | | | | | **№ of patients** | | **Effect** | | **Certainty** | **Importance** |
| --- | --- | --- | --- | --- | --- | --- | --- | --- | --- | --- | --- | --- |
| **№ of studies** | **Study design** | **Risk of bias** | **Inconsistency** | **Indirectness** | **Imprecision** | **Other considerations** | **Usual care** | **placebo** | **Relative (95% CI)** | **Absolute (95% CI)** |  |  |
| **Aerobic_cognitive function** | | | | | | | | | | | | |
| 5 | randomised trials | not serious | serious | not serious | serious | none | 175 | 212 | - | SMD **0.8 higher** (0.23 higher to 1.37 higher) | ⨁⨁◯◯ Low |  |
| **Resistance_cognitive function** | | | | | | | | | | | | |
| 4 | randomised trials | serious | not serious | not serious | serious | none | 122 | 113 | - | SMD **0.36 higher** (0.04 lower to 0.77 higher) | ⨁⨁◯◯ Low |  |
| **Combined_cognitive function** | | | | | | | | | | | | |
| 6 | randomised trials | serious | serious | not serious | not serious | none | 205 | 217 | - | SMD **0.69 higher** (0.31 higher to 1.07 higher) | ⨁⨁◯◯ Low |  |
| **Resistance_muscle strength** | | | | | | | | | | | | |
| 3 | randomised trials | serious | not serious | not serious | serious | none | 58 | 59 | - | SMD **0.79 higher** (0.42 higher to 1.17 higher) | ⨁⨁◯◯ Low |  |
| **Combined_muscle strength** | | | | | | | | | | | | |
| 3 | randomised trials | serious | serious | not serious | serious | none | 134 | 146 | - | SMD **0.82 higher** (0.32 higher to 1.32 higher) | ⨁◯◯◯ Very low |  |
| **Aerobic_physical performance** | | | | | | | | | | | | |
| 5 | randomised trials | not serious | very serious | not serious | serious | none | 175 | 212 | - | SMD **0.56 higher** (0.16 lower to 1.29 higher) | ⨁◯◯◯ Very low |  |
| **Resistance_physical performance** | | | | | | | | | | | | |
| 4 | randomised trials | serious | serious | not serious | serious | none | 126 | 120 | - | SMD **1.03 higher** (0.38 higher to 1.67 higher) | ⨁◯◯◯ Very low |  |
| **Combined_physical performance** | | | | | | | | | | | | |
| 6 | randomised trials | serious | serious | not serious | not serious | none | 204 | 210 | - | SMD **0.91 higher** (0.39 higher to 1.43 higher) | ⨁⨁◯◯ Low |  |


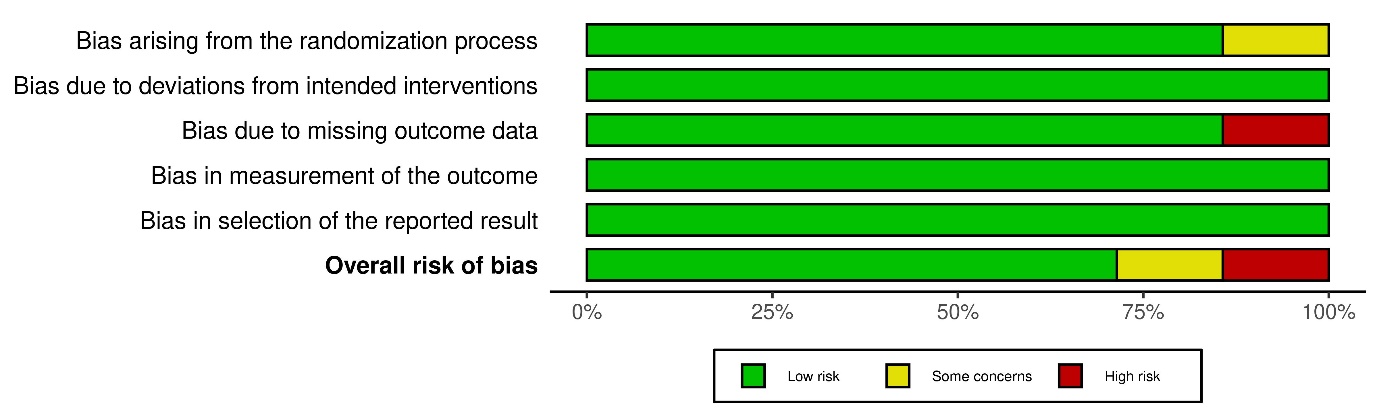


(A) Intention-to-treat


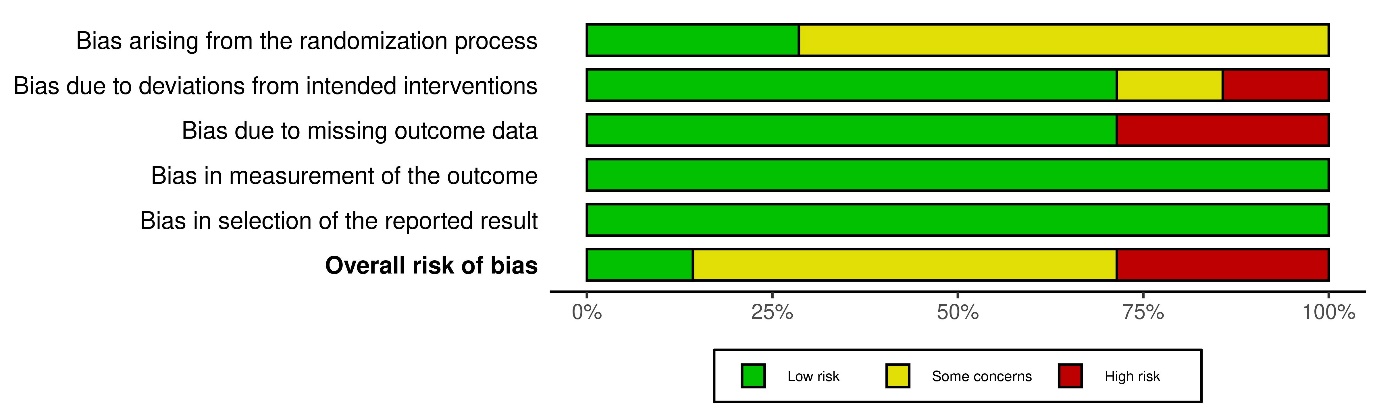


(B) Per-protocol

**Figure S1.** Risk of bias assessment according to ITT and PP analyses
